# Functional Characterization of *VvSK* Gene Family in Grapevine Revealing Their Role in Berry Ripening

**DOI:** 10.3390/ijms21124336

**Published:** 2020-06-18

**Authors:** Jingjue Zeng, Muhammad Salman Haider, Junbo Huang, Yanshuai Xu, Tariq Pervaiz, Jiao Feng, Huan Zheng, Jianmin Tao

**Affiliations:** 1 College of Horticulture, Nanjing Agricultural University, Nanjing 210095, China; 2016204001@njau.edu.cn (J.Z.); salman.hort1@gmail.com (M.S.H.); 2018804197@njau.edu.cn (J.H.); 2018204007@njau.edu.cn (J.F.); taojianmin@njau.edu.cn (J.T.); 2 College of Horticulture, Hunan Agricultural University, Changsha 410000, China; yanshuaixu56@gmail.com; 3 Advance innovation center for tree breeding, Beijing Forestry University, Beijing 100083, China; tariqzoqi2009@gmail.com

**Keywords:** GSK3, grapes, evolutionary analysis, light response, photosynthesis, ripening

## Abstract

The glycogen synthase kinase 3/shaggy kinase (GSK3) is a serine/threonine kinase that plays important roles in brassinosteroid signaling, abiotic stress responses, cell division, and elongation, etc. In this study, we characterized seven grape GSK3 genes, showing high similarities with homologs from other species including *Arabidopsis*, white pear, apple, orange, and peach. Gene chip microarray data derived from an online database revealed very diverse developmental and tissue-specific expression patterns of *VvSKs*. *VvSK3* and *VvSK7* showed much higher expression levels in almost every tissue compared with other members. *VvSK7* was highly enriched in young tissues like berries before the veraison stage, young leaves and green stems, etc., but immediately downregulated after these tissues entered maturation or senescence phases. Prediction of cis-elements in *VvSK* promoters indicated that *VvSKs* might be sensitive to light stimulation, which is further confirmed by the qPCR data. Constitutive overexpression of *VvSK7* in *Arabidopsis* leads to dwarf plants that resembles BR-deficient mutants. The photosynthetic rate was significantly reduced in these plants, even though they accumulated more chlorophyll in leaves. Transient overexpression of *VvSKs* in tomatoes delayed the fruit ripening process, consistent with the observation in grapevine which blocks *VvSKs* by EBR- or BIKININ-promoted berry expansion and soluble solids accumulation. Data presented in the current study may serve as a theoretical basis for the future application of BRs or related compounds in quality grape production.

## 1. Introduction

Glycogen synthase kinase 3 (GSK3) is a highly conserved serine/threonine kinase, implicated in many vital signal transduction pathways in eukaryotes. It was initially found in mammals, having two forms of GSK3, namely, GSK3α and GSK3β, which are described as key enzymes involved in glycogen metabolism [1,2]. Later studies discovered that GSK3 homologs also play crucial roles in various physiological processes in animals, such as protein synthesis, tumorigenesis, regulation of transcription factor activity, determination of cell fate, and glycogen metabolism, etc. [3,4,5,6]. Additionally, GSK3 is an essential component in the animal Wnt signaling pathway [7,8].

Plant *GSK3s* have more homologs with much diverse functions compared to the corresponding genes in animal. In plants, *GSK3s* function as negative regulators in brassinosteroid (BR) signaling. *GSK3s* phosphorylate and inhibit the transcription factors BZR1 and its homolog BES1, which control the BR-responsive gene expression. Perception of BRs through the receptor complex BRI1-BAK1 leads to deactivation and degradation of *GSK3s,* thus alleviating their inhibition to BZR1/BES1 and inducing BR-responsive gene expression [9,10]. Through regulation of downstream BR signaling or direct binding and phosphorylation of key components in other biological pathways, plant *GSK3s* are involved in diverse biological processes, including flower and reproductive organ development, stomatal development and stress responses, etc. In *Arabidopsis*, *AtSK11* and *AtSK12* showed specific strong expression in early floral meristems and restricted to sepal primordia, petals, carpels and the pollen-containing regions of the anthers in the later stage. Antisense reduction in either *AtSK11* or *AtSK12* transcript levels results in disrupted cell division in the floral meristem and leads to abnormal sepals, petals, and carpel development. *AtSK31* may also involve in reproductive organ development as *AtSK31* proteins highly enriched in gametophytes, floral organs, and embryos [11,12,13]. *Arabidopsis AtSK21* directly phosphorylate and inhibit MAPKKK YODA (YDA) and MKK4/MKK5, which are the key components of the MAPK signaling that controls stomatal development and patterning [14]. Interestingly, *AtSK21* also phosphorylates and inhibits transcriptional factor SPCH, which controls the initiation of stomatal development [15]. Work in several plant species has implicated *GSK3s* in abiotic stress responses. Transcripts of multiple *GSK3s* from *Arabidopsis*, rice, and wheat can be induced under salt stress conditions [2]. Overexpressing *AtSK22* in *Arabidopsis* plants induced a marked upregulation of several salt stress-responsive genes, even in the absence of high salinity [16]. In rice, overexpressing *OsGSK5* increased salinity tolerance in part via priority carbon allocation to root starch [17]. Given the dual roles of BR in plant development, *GSK3s* may involve in more diverse and general biological processes that need to be elucidated in the future.

The grape is one of the world-wide largest fruit crops. Studies of *GSK3s* in the grape are very limited even though brassinosteroid-like hormones have already been commercially used in grape production for decades. In the previous studies, only one grape GSK3 gene *VvSK1* was identified and characterized. *VvSK1* was strongly expressed at the post-veraison stage of berries, when the berries start to accumulate glucose, fructose and abscisic acid. Overexpressing *VvSK1 in* grapevine cells highly induced the expression of four monosaccharide transporters, namely, *VvHT3*, *VvHT4*, *VvHT5*, and *VvHT6,* and significantly promoted glucose and sucrose accumulation [18]. Considering *VvSKs* may involve in the regulation of key flavor and quality traits in grape berries, and the wide application of brassinosteroid-like hormones in grape production, systematic identification and characterization of gape *GSK3s* is necessary and of great importance for grape researchers and farmers.

In the present study, we employed bioinformatics approaches to identify and characterize grape *GSK3s* on a genome-wide scale based on several publicly available database. Furthermore, we investigated their spatiotemporal expression profiles in different tissues and developmental stages, by which we selected *VvSK7* as a study candidate to further elucidate the putative roles of *VvSKs* in plant development by constitutively over-expression in *Arabidopsis* plant. These comprehensive results will provide an insight for further studies and assist in a better understanding of the potential functions of *VvSKs* in grapevine.

## 2. Results

### 2.1. Phylogenetic and Conserved Structural Analysis of VvSKs

**Figure 1:**
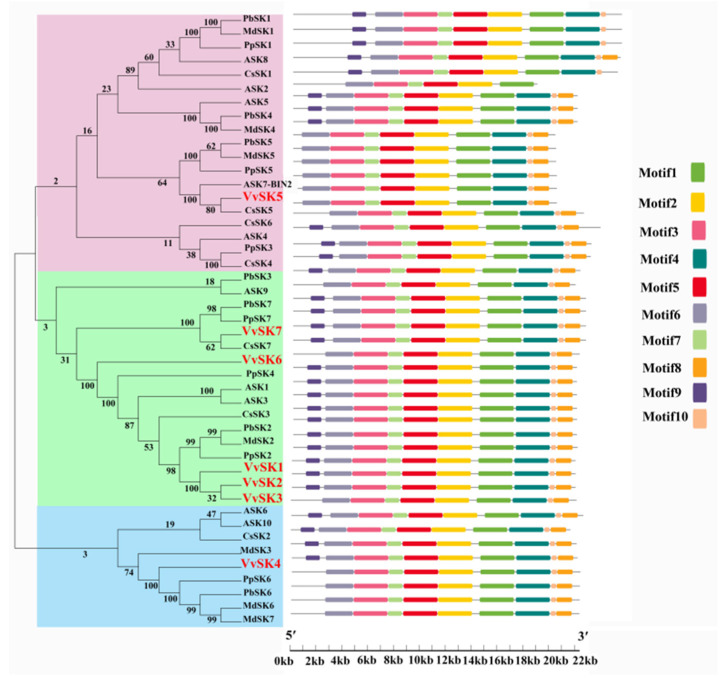
The phylogenetic tree and conserved motif analysis of *GSK* genes. *SKs* from *Vitis vinifera* (red mark), *Arabidopsis thaliana*, *Pyrus bretschneideri*, *Prunus persica*, *Malus domestica and Citrus reticulata* were put together for comparison. The phylogenetic tree was generated by MEGA 6.0 using the maximum likelihood method (time tree). The axis numbers mean the relative divergence time. Ten motifs named motif 1–10 were analyzed using the online program “MEME”.

There are 7 *VvSK* genes named *VvSK1*, *VvSK2*, *VvSK3*, *VvSK4*, *VvSK5*, *VvSK6* and *VvSK7* that were reported in the grapevine genome [18]. The basic information, including gene ID, genomic DNA size, amino acid numbers, and theoretical PI are listed in Table S1. The phylogenetic tree was constructed using *SK* coding sequences (CDS) from Arabidopsis and different fruit species including grape, pear, apple, and peach, etc. (Figure 1). According to the relative divergence times, grape *SK* members are clustered into three subgroups (relative divergence times were higher than 150). *VvSK5* and *VvSK4* are clustered into subgroup I and subgroup III, respectively, while most of the *VvSKs*, including *VvSK1*, *VvSK2*, *VvSK3*, *VvSK6* and *VvSK7,* are clustered into subgroup II. GSK genes from different fruit species are closely correlated. Overall, grape *SKs* showed a closer evolutional relationship with *GSKs* from citrus and peach. *VvSK5* and *VvSK7* are closely clustered with *CsSK5* and *CsSK7* respectively. *VvSK1*, *VvSK2*, and *VvSK3* showed relatively close kinship with *PpSK2*, and *VvSK4* showed relatively close kinship with *PpSK5* and *PpSK6*. (Figure 1). Conserved motif prediction revealed that *SKs* from different species exhibited high similarity in motif composition. Except for motifs 8 and 9, most of the motifs are found in all analyzed *SKs*, indicating the high conservation in protein structures among these *SKs* (Figure 1, Figure S1).

### 2.2. Syntenic Analysis of VvSKs

**Figure 2:**
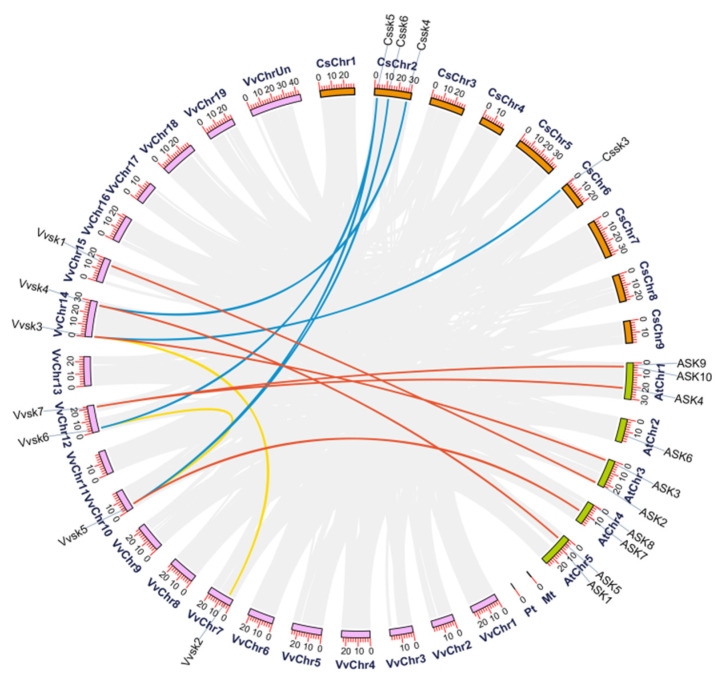
Syntenic block among *SK* genes from *V. vinifera*, *A. thaliana*, and *C. sinensis*. Links between *V. vinifera* and *C. sinensis* are colored in blue, *V. vinifera* and *A. thaliana* in orange, *V. vinifera* and *V. vinifera* in yellow. Chromosomes of *V. vinifera*, *A. thaliana*, and *C. sinensis* were respectively colored in pink, green and orange.

In order to analyze collinearity of *SK* genes in different species, syntenic analysis of *SKs* from *A. thaliana*, *V. vinifera*, and *C. sinensis* was performed. There are 13 collinear gene pairs were generated among grapevine, citrus, and *Arabidopsis* plants (Figure 2, Table S2). The result suggests that *VvSK1* and *ASK2* might share a common ancestor. *VvSK2* originated together with *VvSK3*, and *VvSK3* might have the same origin with *CsSK3* and *ASK3. VvSK4* might share the same ancestor with *CcSK4* and *ASK5. VvSK5* might have the same ancestor with *CsSK5*, *CsSK6*, *ASK7* and *VvSK6*. *VvSK6* might be derived from a common ancestor with *CsSK5*. The origin of *VvSK7* might be same with *ASK4* and *ASK9*. The study of the collinear relationship of *SKs* in these plants showed vital implications for the functional study of *VvSKs*.

### 2.3. Transcriptional Profiling of VvSKs in Different Organs and Developmental Stages in Grapevine

**Figure 3:**
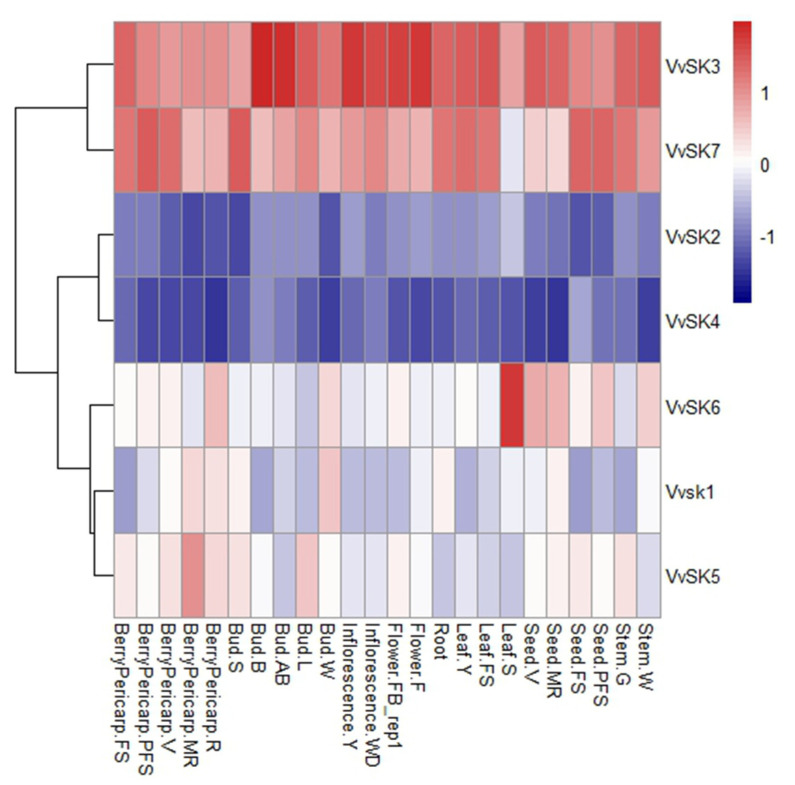
Expression profiles of the *VvSK* genes in different grapevine organs, tissues, and, developmental stages. Data were normalized based on the mean expression values of each gene in all analyzed tissues. BerryPericarp-FS: berry pericarp fruit set; BerryPericarp-PFS: berry pericarp post-fruit set; BerryPericarp-V: berry pericarp veraison; BerryPericarp-MR: berry pericarp mid-ripening; BerryPericarp-R: berry pericarp ripening; Bud-S: bud swell; Bud-B: bud burst (green tip); Bud-AB: bud after-burst (rosette of leaf tips visible); Bud-L: latent bud; Bud-W: winter bud; Inflorescence-Y: a young inflorescence (single flower in compact groups); Inflorescence-WD: well-developed inflorescence (single flower separated); Flower-FB: flowering begins (10% caps off); Flower-F: flowering (50% caps off); Leaf-Y: young leaf (pool of leaves from a shoot of five leaves); Leaf-FS: mature leaf (pool of leaves from a shoot at fruit set); Leaf-S: senescencing leaf (pool of leaves at the beginning of leaf fall); Seed-V: seed veraison; Seed-MR: seed mid-ripening; Seed-FS: seed fruit set; Seed-PFS: seed post-fruit set; Stem-G: green stem; Stem-W: woody stem.

To understand the spatiotemporal expression profiles of *VvSKs*, the global transcriptomic data of developmental phases of 24 different tissues and organs including berry, leaf, flower, stem, root, and seed were retrieved from NCBI (GSE36128) [19]. The results (Figure 3) showed that different *VvSK* members have very diverse developmental and tissue-specific expression patterns. Transcription of *VvSK3* and *VvSK7* were highly expressed in almost every tissue. *VvSK3* showed especially high expression levels in buds and reproductive tissues, including inflorescences and flowers. The expression of *VvSK7* was more diverse in different tissues. Interestingly, this gene showed very high expression levels in most of the young tissues, such as berries (including pericarp and seeds) at fruit set, post-fruit set and veraison stages, leaves before maturation, swelling buds and green stems, but very low expression levels in mature tissues such as ripening berries and senescing leaves. *VvSK2* and *VvSK4* showed very weak expression levels in almost every tissue. *VvSK1*, *VvSK5,* and *VvSK6* showed moderate expression levels in different tissues. *VvSK6* showed complementary expression patterns with *VvSK7,* e.g., *VvSK7* showed very low expression level in senescing leaves while *VvSK6* was highly enriched in this tissue.

### 2.4. Cis-Elements in the Promoter of SK Genes

**Figure 4:**
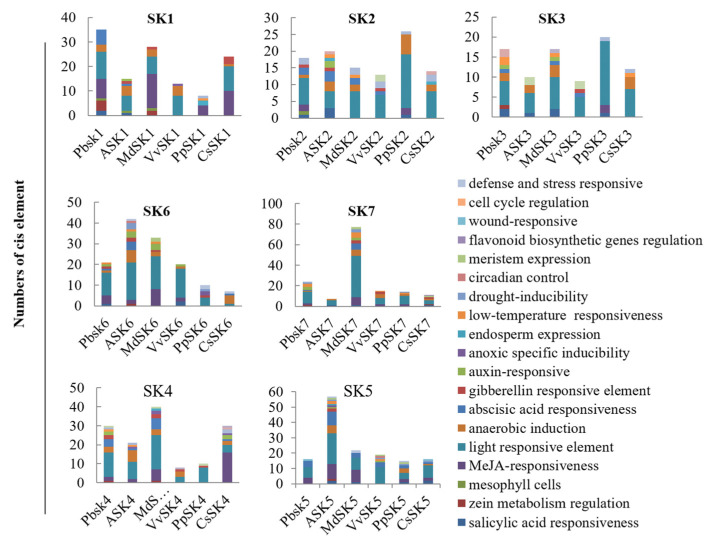
Cis-elements in the promoter regions of *SK* genes. Cis-elements in promoter regions of the SK genes from *Vitis vinifera*, *Arabidopsis thaliana, Pyrus bretschneideri*, *Prunus persica*, *Malus domestica* and *Citrus reticulata*. An upstream of 1500bp of different *SK* genes were selected as promoter regions and applied Cis-elements prediction in online database “PlantCARE”.

To further clarify the gene function and transcriptional regulation mechanism of *SKs*, cis-elements in the promoter region of *SK* genes from different species were predicted using an online database “PlantCARE”. Cis-elements related to light response, MeJA response, anaerobic induction, and gibberellin response were the most abundant ones in these *SKs* promoters (Figure 4). Photoreaction related cis-elements such as Box 4, GATA-motif, GT1-motif, AE-box, G-box, MRE, TCCC-motif and TCT-motif were highly enriched in most of the *SKs* and especially in *VvSKs*, indicating *VvSKs* would be involved in photosynthesis or in light responsive signaling.

### 2.5. VvSKs are Sensitive to Dark Treatment

**Figure 5:**
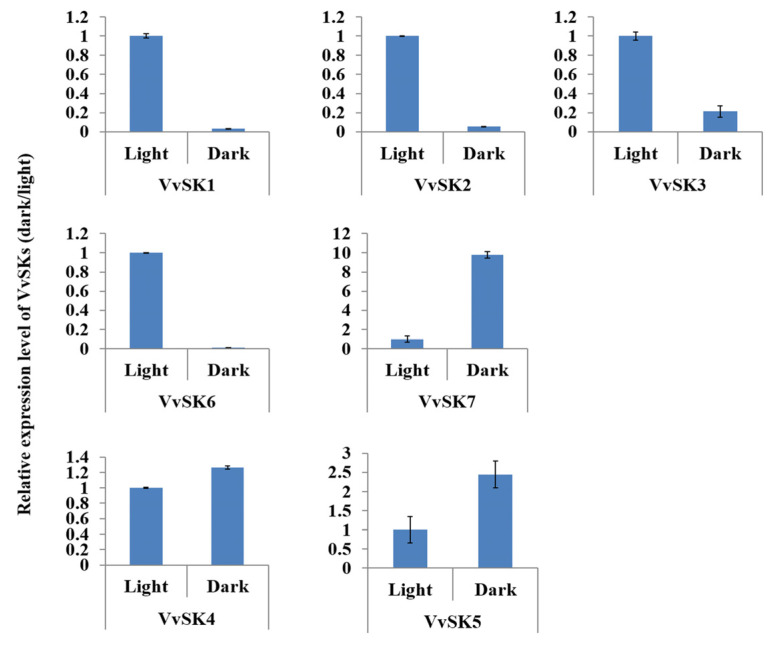
Responsive patterns of *VvSKs* upon dark treatment. *VvSK* gene expression levels in leaves of ‘Shine Muscat’ grape under light and dark treatment were evaluated by qPCR. Housekeeping gene *VvACTIN* was used as an internal reference. Expression levels were normalized with light treatment samples.

Light-responsive cis-elements were highly enriched in promoter regions of *VvSKs* (Figure 4), indicating that these genes could be sensitive to light or dark stimulation. To verify these light-responsive cis-elements, transcriptional levels of *VvSKs* under dark treatment were analyzed. The results (Figure 5) showed that all the *VvSKs* exhibited light-responsive patterns. Expression levels of *VvSKs* in subgroup II including *VvSK1*, *VvSK2*, *VvSK3,* and *VvSK6* dramatically decreased upon 8-h dark treatment. In contrast, expression levels of *VvSKs* in subgroup I (*VvSK5*) and subgroup III (*VvSK4*) were induced upon dark treatment. These contradictory responsive patterns indicate that *VvSKs* in subgroup II may function complementary with *VvSKs* in subgroup I and III in light-related photoreaction or signaling pathways.

### 2.6. Constitutive Over-Expression of VvSK7 Affects BR Signaling and Inhibits Plant Photosynthesis

**Figure 6:**
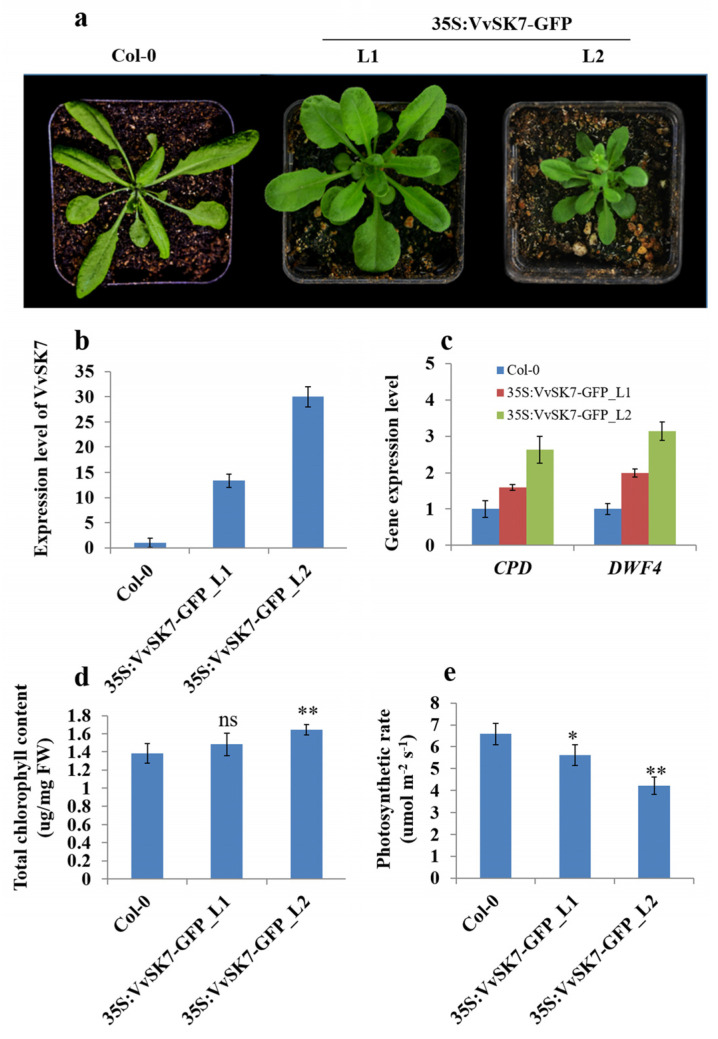
Constitutive over-expression of *VvSK7* affects BR signaling and inhibits photosynthesis. (**a**) Plant phenotype of Col-0, 35s:*Vvsk7*-GFP-L1 and 35s:*Vvsk7*-GFP-L2 at four weeks after germination. (**b**) Expression levels of *VvSK7* in Col-0, 35s:*Vvsk7*-GFP-L1 and 35s:*Vvsk7*-GFP-L2. qPCR experiment was performed using the second true leaf from four-week old plants. Housekeeping gene *AtACTIN* was used as an internal reference. Expression levels were normalized with control Col-0. (**c**) Expression levels of the BR-signaling marker genes of *DWF4* and *CPD*. (**d**) Total chlorophyll content in leaves from four-week old Col-0, 35s:*Vvsk7*-GFP-L1 and 35s:*Vvsk7*-GFP-L2 plants. The second true leaf was used for measurement. (**e**) Photosynthetic rate in the second true leaves from four-week old Col-0, 35s:*Vvsk7*-GFP-L1 and 35s:*Vvsk7*-GFP-L2 plants. In graph d and e, vertical bars represented standard deviations (SD) of means (*n* = 3). The asterisks indicated statistically significant differences compared with Col-0, which were determined by two-tailed Student’s t-test. * Represents *p* ≤ 0.05, ** represents *p* ≤ 0.01, ns represents no significant difference.

To further verify the function of *VvSKs*, constitutive over-expression of *VvSK7* driven by cauliflower mosaic virus promoter (CaMV 35S) in *Arabidopsis* Col-0 was generated. Two individual transgenic lines of L1 and L2 with 13 or 30-folds over-expression of *VvSK7,* respectively, were examined (Figure 6b). Four-week old plants of 35S:*VvSK7*-GFP_L1 showed roundish and compact rosette leaves, and shorter petioles compared with Col-0. 35S:*VvSK7*-GFP_L2 with higher expression of *VvSK7* showed much severe phenotype than L1. Except for the roundish and compact rosette leaves, L2 plants are dwarf and showed stunted growth. These phenotypes resemble *AtSK21* gain of function [20] or BR-deficient mutants like dwf5-7 [21] in *Arabidopsis* or *AtSK* gain of function mutant-like bin2-1 [22] (Figure 6a). Moreover, expression levels of BR-signaling marker genes DWF4 and CPD were all increased greatly in L1 and L2 plants (Figure 6c), indicating that BR signaling was blocked in *VvSK7*-OE plants. Besides, *VvSK7*-OE plants also showed dark green colored leaves. The quantification showed that their total chlorophyll content, especially in the L2 plant, was significantly higher than that in Col-0 plants (Figure 6d). Interestingly, even though chlorophyll was more accumulated in *VvSK7*-OE plants, photosynthetic rate in these plants was reduced compared with Col-0 plants (Figure 6e). These results suggested that the BR hormone could be involved in photosynthesis in grapevine under the modulation of *VvSKs*.

### 2.7. VvSKs Inhibit Fruit Ripening in Tomato

**Figure 7:**
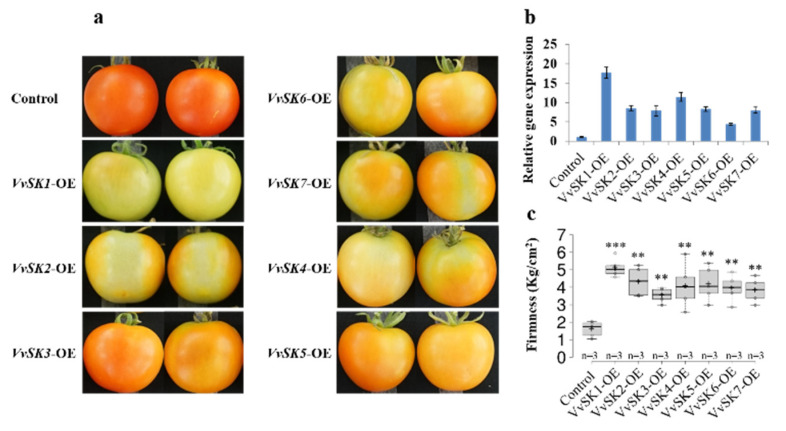
Transient over-expression of *VvSKs* in tomato inhibit fruit ripening. The 35S:*VvSK7*-GFP construct was injected into the fruits of 12 days after flowering, and empty PK7FWG2 was used as a control. (**a**) Tomato fruits 4 days after infiltration, OE: overexpression; (**b**) Transcription levels of *VvSKs* in tomato fruits 4 days after infiltration. Housekeeping gene *SlACTIN* was used as an internal reference. Expression levels were normalized with control (tomato fruits infiltrated with GV3101 Agrobacterium cultures containing pK7FWG2 empty vector); (**c**): Firmness of tomato fruits 4 days after infiltration. Three independent fruits from three replicates were used for measurement (*n* = 3). The asterisks indicated statistically significant differences compared with control, which were determined by two-tailed Student’s t-test. * Represents *p* ≤ 0.05, ** represents *p* ≤ 0.01, *** represents *p* ≤ 0.001.

The BR hormone is involved in fruit ripening in strawberry [23], but whether this is also the case in grapevine and whether this process is mediated by *VvSKs* is yet to be verified. In order to corroborate these questions, *Agrobacterium tumefaciens* strain GV3101 cultures containing 35S: *VvSKs*-GFP or the empty vector pK7FWG2 (control) were infiltrated into mature green tomato fruits of *Lycopersicon esculentum* cv. Moneymaker. Tomato fruits were characterized 4 days after infiltration. Real-time PCR results showed that transcript levels of *VvSKs* showed at least a 5-fold upregulation compared with the control fruits 5 days after infiltration (Figure 7b). The surface color of control fruits developed into fully red just 5 days after infiltration, while the changes in surface color from green to red were markedly delayed in the 35S: *VvSK*-GFP fruits, especially in 35S: *VvSK1*-GFP fruits, which only turned slight yellow 4 days after infiltration (Figure 7a). In addition, firmness of 35S: *VvSK*-GFP fruits was also much higher compared with that of control fruits (Figure 7c). Altogether, these results indicated that *VvSKs* inhibit fruit ripening in tomatoes.

### 2.8. BR Promotes Grape Berry Expansion and Soluble Solids Accumulation

**Figure 8:**
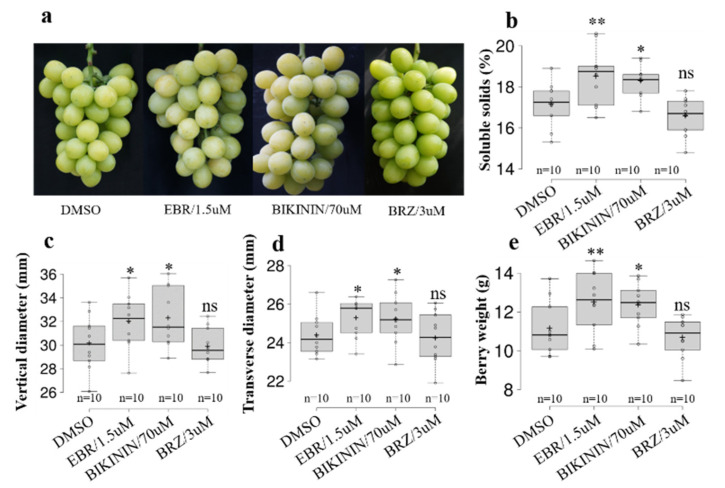
Effect of EBR, BIKININ and BRZ on grape berry development. Berries were treated at veraison stage. Imaging and measurement of quality-related parameters were performed at maturity stage: (**a**) Berry phenotypes upon DMSO,EBR,BIKININ or BRZ treatment; (**b**) Soluble solids of grape berries upon DMSO,EBR,BIKININ or BRZ treatment; (**c**) Longitudinal diameter of grape berries upon DMSO,EBR,BIKININ or BRZ treatment; (**d**) Transverse diameter of grape berries upon DMSO,EBR,BIKININ or BRZ treatment; (**e**) Grape berry weight upon DMSO,EBR,BIKININ or BRZ treatment. For each treatment, 10 representative berries from 3 individual grape clusters were used for measurement (*n* = 10). The asterisks indicated statistically significant differences compared with the mock treatment (DMSO), which were determined by two-tailed Student’s t-test. * Represents *p* ≤ 0.05, ** represents *p* ≤ 0.01, ns represents no significant difference.

Over-expression of *VvSK7* inhibited photosynthesis in Arabidopsis and transient expression of *VvSKs* in the tomato delayed fruit ripening in it. These findings provided a strong indication for the involvement of *VvSKs* and their corresponding BR hormones in grape berry development. In order to figure out this question, 24-epibrassinolide, brassinazole (biosynthesis inhibitor of brassinosteroid) and BIKININ (GSK3/Shaggy-like kinase inhibitor) were applied to grape berries of the Shine Muscat (*Vitis labruscana Bailey*× V. *vinifera L*. Shine Muscat) variety at veraison stages. Fruit quality-related parameters including fruit size, berry weight, and soluble solids were measured at maturity (17 August). Compared with the mock treatment, EBR and BIKININ all promoted grape berry ripening, the surface color of EBR- and BIKININ-treated berries turned white just 2 weeks after treatment, while the berries treated with BRZ (brassinazole) remained green (Figure 8a). Moreover, EBR- and BIKININ-treated berries significantly accumulated more soluble solids than the mock berries (Figure 8b). Furthermore, EBR and BIKININ also promoted grape berry expansion in both vertical and transverse diameters, and the weight of their berries was significantly higher than the mock and BRZ-treated berries. Taken together, these results demonstrated that EBR and BIKININ can promote grape ripening and berry expansion, which is consistent with the result that transient over-expression of *VvSKs* in tomatoes delayed fruit ripening. In addition, *VvSK7* is a negative regulator for photosynthesis, thus, inhibition of *VvSKs* by applying EBR and BIKININ can promote photosynthesis and accelerate grape berry expansion and soluble solids accumulation.

## 3. Discussion

Protein kinases form a large family of enzymes that mediate eukaryotic cell responses to external stimuli [24]. To date, several protein kinase family members have been identified, including GSK3, Pto-like protein kinase, MAPKKK and PP2C [25,26,27,28]. In these kinases, GSK3 is reported to regulate different physiological and developmental processes in mammals and plants [2,7,29,30,31]. However, the research of GSK3-like kinases has been limited to several model plants. Recent studies have shown that the GSK3 family or their corresponding BR hormones have been emerging as an important genetic engineering targets or plant growth regulators in numerous crops like rice (*Oryza sativa*) [17,32] and maize (Zea mays. L) [33]. In grapevine, GSK3 homolog *VvSK1* is reported as a sugar-inducible protein kinase that regulates hexose transport and sugar accumulation in cultured grapevine cells [18]. In contrast, their evolutional relationship between different fruit crops and their function in grape berry development has not been elucidated for decades.

In the current study, a phylogenetic tree using *VvSKs* and *GSK* homologs from other fruit crops was constructed, and conserved motif prediction and syntenic analysis were also carried out. These results suggested that *GSK* genes in different fruit crops are highly conserved as they shared similar motif composition. *VvSKs* are evolutional closer to *GSK* genes in citrus and peach crops.

BRs are reported to be involved in light-regulated processes [34]. The BR-signaling transcriptional factor BZR1 represses photoreceptors phytochrome B and phototropin 1 and induces photo-morphogenesis negative regulators including CONSTITUTIVE PHOTOMORPHOGENEIC 1 (COP1) and SUPPRESSOR OF PHYTOCHROME A (SPA1), leading to defects in plant photo-morphogenesis [35]. The BR hormone is also involved in photosynthesis by modulation of *ϕ*PSII (efficiency of PSII) [36,37] and rubisco activity [38]. The current study suggested that light-responsive cis-element was highly enriched in promoter regions of *VvSKs*, Moreover, qPCR results also indicated that *VvSKs* are sensitive to the light response. Collectively, these findings suggested that *VvSKs* could mediate a feedback regulation of BR signaling in light-regulated processes. On the one hand, the BR-signaling downstream transcriptional factor BZR1 directly regulate photomorphogenesis or photosynthesis by repressing or inducing important components in these processes. On the other hand, light signaling can in turn alleviate or enhance its effect by modulation of *VvSK*-mediated phosphorylation and inhibition of BZR1 [39].

*VvSK7* was highly enriched in the young tissues such as berries before the veraison stage, young leaves, and green stems, etc. Interestingly, *VvSK7* was immediately downregulated once these tissues entered maturation or senescence processes, its expression level showed obvious downregulation in ripening berries and senescencing leaves, as shown in Figure 3. This observation provides a strong indication that *VvSK7* is important for early tissue development but could be negative for maturation processes. Constitutive overexpression of *VvSK7* in *Arabidopsis* lead to weakened BR signaling and defect in plant growth, which is consistent with its homologs in *Arabidopsis* [22,39]. In addition to the BR-related phenotype, *VvSK7*-OE plants showed more chlorophyll accumulation in leaves, but lower photosynthetic rate compared to Col-0 (Figure 6d,e), which is inconsistent, as its commonly assumed that chlorophyll content is positively correlated with photosynthesis rate [40,41]. Another observation of Rubisco and Rubisco activase could explain this inconsistency, as we also found that Rubisco activity in *VvSK7*-OE plants was decreased when compared to Col-0 and a putative interaction between Rubisco activase (RCA) and *VvSK7* (data not shown). Over-expression of *VvSK7* could inhibit Rubisco activity through RCA and lead to lower photosynthesis efficiency in *Arabidopsis* [42,43]. A similar study in *C. sativus* showed that Rubisco activity and photosynthesis rate were enhanced upon brassinosteroid treatment, which is consistent with our hypothesis [37]. Transiently overexpression of *VvSKs* in the tomato lead to delayed fruit ripening (Figure 7), consistent with the assumption that *VvSK7* could be a negative regulator for maturation. Brassinosteroids were found to be involved in strawberry ripening. BR hormone content and transcriptional level of the BR-signaling receptor *FaBRI1* were all increased during the ripening process [23]. By contrast, we observed the downregulation of *VvSK7* when the grape berry entered the ripening process; however, the exact role and the underlying molecular mechanism still need to be elucidated.

In this study, we applied 24-epibrassinolide, BIKININ (GSK3/Shaggy-like kinase inhibitor) and brassinazole (biosynthesis inhibitor of brassinosteroid) at the veraison stages. The results showed that EBR and BIKININ treatment accelerated grape berry ripening, promoted fruit expansion, and soluble solids accumulation. BR treatment leads to dephosphorylation and proteasome-mediated degradation of *GSKs* [44], while small molecular compound BIKININ directly blocks the GSKs’ kinase activity though competing for the ATP binding pocket [45]. Blocking *VvSKs* in the grape berry by these compounds promotes berry ripening, which is consistent with the observation in the tomato where fruit ripening was delayed by overexpression of *VvSKs*. Previous studies reported that overexpression of *VvSK1* in cultured grapevine cells leads to upregulation of monosaccharide transporters *VvHT3*, *VvHT4*, *VvHT5*, and *VvHT6* and results in increased glucose and sucrose accumulation [18]. However, different results are observed in the current study, showing that blocking *VvSKs* by EBR and BIKININ promoted the accumulation of soluble solids in berries. Considering that overexpression of *VvSK7* leads to weakened photosynthesis rate, blocking *VvSK7* by EBR or BIKININ will, in turn, promote photosynthesis which will ultimately lead to the accumulation of soluble solids in berries. Moreover, the expression level of *VvSK7* in berries was significantly higher than that of *VvSK1* (Figure 3), which means that the positive effect of EBR or BIKININ through *VvSK7* could cover or compensate their negative effects through *VvSK1.*

## 4. Materials and Methods

### 4.1. Plant Materials and Treatments

One-year-old potted Shine Muscat grapevine seedlings grown in the greenhouse at Nanjing Agricultural University, Nanjing, Jiangsu, China were used for dark/light treatment. Before treatment, the grapevine seedlings were transferred to a plant growth chamber at 25 °C with continuous light (5000 lx). Leaves at 4–6 nodes were used for treatment. Along the central vein, half of the leaf was covered with tinfoil paper as a dark treatment, the other half was exposed to the light as light treatment. The samples were harvested 8 h after treatment. The collected leaves were immediately frozen in liquid nitrogen and stored at −80 °C.

Five-year-old Shine Muscat Grapevines were grown in Tangshan Grape Test Base at Nanjing (N32°2′27″, E118°59′57″), Jiangsu Province, China. Grapevines with similar growth conditions were selected for compound treatments. Water containing 1.5 umol/L EBR (CAS No. 78821-43-9, Yuanye, Shanghai, China), 70 umol/L BIKININ (CAS No. 188011-69-0, Yuanye, Shanghai, China), 3 umoL/L BRZ (CAS No. 224047-41-0, Yuanye, Shanghai, China) or DMSO (mock control, CAS NO. 67-68-5, Aladdin) was sprayed evenly on the grape berries using a 250 mL hand-held sprayer. Treatments were applied every 2 weeks from veraison stage (26 June) to mature stage (17 August). For each treatment, 10 representative fruits from 3 individual fruit clusters were detached at maturity for physiological parameter measurement.

Transgenic Arabidopsis plants used in this study were grown in a plant growth chamber at 22 °C under a 16 h-light/8 h-dark cycle.

Tomato plants (*Lycopersicon esculentum* cv. Moneymaker) prepared for *VvSKs* transient over-expression assay were grown in a greenhouse at Nanjing Agricultural University, Nanjing, Jiangsu, China.

### 4.2. Gene Cloning and Construction of the Expression Vectors

*VvSK* gene sequence was queried from BLAST on NCBI. The specific primers were designed for PCR amplification (Table S3) and PCR amplification was conducted as follows: at 95 °C predenaturation for 5 min, 95 °C for 10 s, 55 °C for 15 s, 68 °C for 2 min, 35 cycles, and 68 °C for 10 min extension. All primers were subjected to normal PCR and the reaction products were separated on a 1% agarose gel to ensure the bands were of the expected size and that there were no primer dimers. The target fragment of PCR run-off product was purified, recovered, and linked to the entry vector PDONR221. It was subsequently subcloned into Gateway ready vector PK7FWG2 to generate 35S:*VvSKs*-GFP plant expression vector using the LR reaction of Gateway recombination-based cloning (Invitrogen, Carlsbad, CA, USA). The positive clones were screened and sent for sequencing to the biological engineering company, Shanghai, China.

### 4.3. Transgenic Arabidopsis Method

For gene transformation, a DNA construct was transformed into 5-week-old Arabidopsis using the Agrobacterium tumefaciens strain C58C1 (Weidi, Shanghai, China) and the floral-dip method [46]. Transformed seeds were selected on Murashige and Skoog agar medium containing the appropriate antibiotics: 50 μg/mL kanamycin (Sigma).

### 4.4. Tomato transient expression

Using the freeze–thaw method, 35S:*VvSKs*-GFP vectors were transformed into the Agrobacterium strain GV3101(Weidi, Shanghai, China) [47]. About 5 mL culture of each strain was grown overnight at 28 °C in Luria–Bertani (LB) medium (50 mg/mL kanamycin and 50 mg/mL rifampicin, 10 mM MES, 20 mL acetosyringone). The overnight cultures were inoculated into 50 mL of LB medium and grown at 28 °C overnight. The cells were harvested by centrifugation (3000× *g*, 5 min, 20 °C) and suspended in infiltration buffer (10 mM MgCl_2_, 10 mM MES, 20 mL acetosyringone), adjusted to an optical density (OD600) of 0.8–1.0 of PK7WG2 and its derivatives, and left to stand at room temperature for 4 h. About 1 mL of Agrobacterium was infiltrated into every tomato at mature green stage with a 1 mL syringe. Ten uniformly sized fruit was used in the infiltration experiment, and the experiment was repeated three times.

### 4.5. Determination of Photosynthetic Rate

Li-6800 (LI-COR, Lincoln, NE, USA) portable photosynthetic instrument was used to measure the photosynthetic rate. The second true leaf from 4-week old *Arabidopsis* plants were used for measurement. 3 leaves from 3 individual plants were measured. The measured temperature is (20 ± 2) °C, and the light intensity is 1000 µmol·m^−2^·s^−^^1^. The relative humidity is 25–30%, the concentration of CO_2_ is (380 ± 10) µmol·mol^−1^, the flow rate is 400 µmol·s^−^^1^, and the average value of three readings is taken as the measured value for each leaf.

### 4.6. Chlorophyll Measurement

Use a punch to cut about 1 cm^2^ of leaves (avoiding relatively thick veins) and cut about 5 mm long and 1 mm wide filaments. Put the leaf filaments into the calibration tube containing 5 mL 80% acetone, and place in the dark after sealing the nozzle until the filaments are completely white (overnight). Pour the solution into the cuvette and use the two wavelengths of 662 nm and 645 nm as the peak absorbencies of chlorophyll-a and chlorophyll-b. Calculate the chlorophyll concentration (CV = chla + chlb) of the extract according to the formula of Arnon [48].

### 4.7. Determination of Fruit Quality

Ten representative fruits from 3 individual grape clusters were picked in each treatment at the fruit mature stage. The vertical and horizontal meridians of the fruits were measured by vernier caliper; the soluble solid content of the fruits was measured by a hand-held sugar meter(PAL-1, Atago, JPN); the titratable acid content was measured by acid-base titration.

### 4.8. Genome-Wide Identification and Annotation of Grape SKs Genes

We quoted seven *VvSKs* from a paper written by Fatma Lecourieux, and using *VvSKs* as a template, we found other *Sks* in *Pyrus bretschneideri, Malus domestica, Citrus reticulata* and *Amygdalus persica* L. All the obtained sequences were stored in the InterProScan database (http://www.ebi.ac.uk/Tools/pfa/iprscan5/, accessed on 5. 7. 2019). Length of sequences, molecular weights and isoelectric points of deduced polypeptides were calculated by using tools provided at the ExPasy website (http://web.expasy.org/protparam/, accessed on 20. 7. 2019) [49]. The choice of candidate *SKs* in *Prunus persica*, *Malus domestica*, *Pyrus bretschneideri*, *Citrus sinensis* was also based on the E-value (1 × 10^−5^) and the highest similarity scores to *VvSKs* in NCBI. All the names and GenBank accession numbers of *SKs* are shown in Table S4.

### 4.9. Phylogenetic Tree, Conserved Motifs, Syntenic Analysis, Transcriptional Profiling and Cis-Elements Analysis of SKs Family

MEGA version 6 (Sudhir Kumar, Arizona State University, Temp, AZ, USA) was used to construct phylogenetic trees by the maximum likelihood (ML) methods and the bootstrap test carried out with 1000 replicates [50]. The conserved motifs were identified using the online MEME program (version 4.12.0) [20]. We set the motif number as 10 and chose motifs with E-values ≤ 1 × 10^−30^. MCscanX was used to analyze the gene synteny and collinearity of *VvSKs* among *Vitis vinifera*, *A. thaliana* and *C. sinensis* [51]. The synteny figures were drawn by Circos-0.69 [52] and those results with E-value > 1 × 10^−5^ were filtered. Global transcriptomic data retrieved from NCBI (GSE36128) were used to generate the heatmap. The heatmap was constructed using R with “pheatmap” package (https://cran.r-project.org/web/packages/pheatmap/index.html, accessed on 10. 3. 2020) [19]. The 1500 bp upstream of the *SK* genes of each species was used to perform cis-elements analysis in PlantCARE [53].

### 4.10. RNA Isolation and RT-PCR Analysis

Total RNA of grape tissue was extracted with the plant total RNA isolation Kit Plus from Fuji (Chengdu, China) according to the manufacturer’s protocol. The concentration of total RNA was measured using a NanoDrop 2000 UV-Vis spectrophotometer (Thermo Scientific, Waltham, MA, USA) after treatment of genomic DNA with RNase-free DNase I (Takara, Dalian, China). The PrimeScriptTM RT reagent Kit (Takara) was used to obtain cDNA according to the manufacturer’s instructions. Synthesized cDNA concentration was diluted to 100 ng/µL and each reaction mixture contained 10.0 µL SYBR Premix Ex Taq TM (Takara), 0.5 µL of each primer (10 µM), 1 µL cDNA, and 8 µL ddH2O in a total volume of 20 µL. Reactions were performed under the following conditions: 95 °C preheating for 4 min, followed by 40 cycles at 95 °C for 20 s, 60 °C for 20 s, and 72 °C for 40 s. The primer pairs (shown in Table S3) were designed by Primer 3 (http://primer3.ut.ee/, accessed on 20. 5. 2019). The PCR experiment was carried out with at least three technical replicates. The relative transcript levels of selected genes were calculated using the 2^−ΔΔ*C*t^ method [54].

### 4.11. Statistical Analysis 

Significant differences mentioned in Figure 6 (chlorophyll content and photosynthetic rate of 35 s: *Vvsk7*-GFP-L1 and L2 compared with that of Col-0), Figure 7 (firmness of *VvSK* transient over-expression tomatoes compared with that of control) and Figure 8 (physiological parameters of EBR-, BIKININ- or BRZ- treated grape berries compared with that of DMSO-treated grape berries) were analyzed by two-tailed Student’s t test using software SAS 9.2 (SAS Institute Inc., Cary, NC, USA). The chart was performed by Excel 2010. 

## 5. Conclusions

We identified seven *VvSK* genes through bioinformatics analysis, and found that *Vvsks* are highly conserved with the homologous genes of other species. The photoreceptor-related elements in the *VvSK* promoter region are the most abundant, and the transcriptional levels of *Vvsk7* were highly enriched in young tissues, but decreased rapidly with their maturation or aging. In addition, the over-expression of phenotype of *Vvsk7* in *VvSKs* was similar to that in BR-deficient Arabidopsis. More chlorophyll was accumulated in the leaves of *VvSK7*-OE plants, but its photosynthetic efficiency was lower than that of wild type plants. Furthermore, transient expression of *Vvsks* delayed the ripening of the tomato fruit. In the grape, the application of EBR or BIKININ might block the activity of *Vvsks* and promote ripening processes such as fruit expansion and soluble solid accumulation. These findings could lay the theoretical foundation for the functional study of *SKs* and the further construction of grape BR regulation networks.

## Supplementary Materials

Supplementary materials can be found at https://www.mdpi.com/1422-0067/21/12/4336/s1.

## References

[1] Yoo M.J., Albert V.A., Soltis P.S., Soltis D.E. (2006). Phylogenetic diversification of glycogen synthase kinase 3/SHAGGY-like kinase genes in plants. BMC Plant Biol..

[2] Saidi Y., Hearn T.J., Coates J.C. (2012). Function and evolution of ‘green’GSK3/Shaggy-like kinases. Trends Plant Sci..

[3] Simpson P., El Messal M., del Prado J.M., Ripoll P. (1988). Stripes of positional homologies across the wing blade of Drosophila melanogaster. Development.

[4] He X., Saint-Jeannet J.-P., Woodgett J.R., Varmus H.E., Dawid I.B. (1995). Glycogen synthase kinase-3 and dorsoventral patterning in Xenopus embryos. Nature.

[5] Petersen C.P., Reddien P.W. (2009). Wnt signaling and the polarity of the primary body axis. Cell.

[6] Yang X.-G., Liang W.-H., Li F., Ma W.-S. (2012). OsGSK3 is a novel GSK3/SHAGGY-like gene from *Oryza sativa* L., involved in abiotic stress signaling. Pak. J. Bot..

[7] Wu D., Pan W. (2010). GSK3: A multifaceted kinase in Wnt signaling. Trends Biochem. Sci..

[8] Woodgett J.R., Cohen P. (1984). Multisite phosphorylation of glycogen synthase: Molecular basis for the substrate specificity of glycogen synthease kinase-3 and casein kinase-II (glycogen synthase kinase-5). Biochim. Biophys. Acta Protein Struct. Mol. Enzymol..

[9] Li J., Nam K.H. (2002). Regulation of brassinosteroid signaling by a GSK3/SHAGGY-like kinase. Science.

[10] Ryu H., Kim K., Cho H., Park J., Choe S., Hwang I. (2007). Nucleocytoplasmic shuttling of BZR1 mediated by phosphorylation is essential in Arabidopsis brassinosteroid signaling. Plant Cell.

[11] Dornelas M.C., van Lammeren A.A., Kreis M. (2000). Arabidopsis thaliana SHAGGY-related protein kinases (AtSK11 and 12) function in perianth and gynoecium development. Plant J..

[12] Charrier B., Champion A., Henry Y., Kreis M. (2002). Expression profiling of the whole Arabidopsis shaggy-like kinase multigene family by real-time reverse transcriptase-polymerase chain reaction. Plant Physiol..

[13] Tavares R., Vidal J., van Lammeren A., Kreis M. (2002). AtSKθ, a plant homologue of SGG/GSK-3 marks developing tissues in Arabidopsis thaliana. Plant Mol. Boil..

[14] Kim T.-W., Michniewicz M., Bergmann D.C., Wang Z.-Y. (2012). Brassinosteroid regulates stomatal development by GSK3-mediated inhibition of a MAPK pathway. Nature.

[15] Gudesblat G.E., Schneider-Pizoń J., Betti C., Mayerhofer J., Vanhoutte I., Van Dongen W., Boeren S., Zhiponova M., De Vries S., Jonak C. (2012). SPEECHLESS integrates brassinosteroid and stomata signalling pathways. Nat. Cell Bio..

[16] Piao H.L., Lim J.H., Kim S.J., Cheong G.W., Hwang I. (2001). Constitutive over-expression of AtGSK1 induces NaCl stress responses in the absence of NaCl stress and results in enhanced NaCl tolerance in Arabidopsis. Plant J..

[17] Thitisaksakul M., Arias M.C., Dong S., Beckles D.M. (2017). Overexpression of GSK3-like Kinase 5 (OsGSK5) in rice (*Oryza sativa*) enhances salinity tolerance in part via preferential carbon allocation to root starch. Funct. Plant Biol..

[18] Lecourieux F., Lecourieux D., Vignault C., Delrot S. (2010). A sugar-inducible protein kinase, VvSK1, regulates hexose transport and sugar accumulation in grapevine cells. Plant Physiol..

[19] Fasoli M., Dal Santo S., Zenoni S., Tornielli G.B., Farina L., Zamboni A., Porceddu A., Venturini L., Bicego M., Murino V. (2012). The grapevine expression atlas reveals a deep transcriptome shift driving the entire plant into a maturation program. Plant Cell.

[20] Tamura K., Stecher G., Peterson D., Filipski A., Kumar S. (2013). MEGA6: Molecular evolutionary genetics analysis version 6.0. Mol. Boil. Evol..

[21] Du J., Zhao B., Sun X., Sun M., Zhang D., Zhang S., Yang W. (2017). Identification and characterization of multiple intermediate alleles of the key genes regulating brassinosteroid biosynthesis pathways. Front. Plant Sci..

[22] Yan Z., Zhao J., Peng P., Chihara R.K., Li J. (2009). BIN2 functions redundantly with other Arabidopsis GSK3-like kinases to regulate brassinosteroid signaling. Plant Physiol..

[23] Chai Y.-m., Zhang Q., Tian L., Li C.-L., Xing Y., Qin L., Shen Y.-Y. (2013). Brassinosteroid is involved in strawberry fruit ripening. Plant Growth Regul..

[24] Hanks S.K., Quinn A.M., Hunter T. (1988). The protein kinase family: Conserved features and deduced phylogeny of the catalytic domains. Science.

[25] Venkatesh J., Jahn M., Kang B.-C. (2016). Genome-wide analysis and evolution of the Pto-like protein kinase (PLPK) gene family in pepper. PLoS ONE.

[26] Cao J., Jiang M., Li P., Chu Z. (2016). Genome-wide identification and evolutionary analyses of the PP2C gene family with their expression profiling in response to multiple stresses in Brachypodium distachyon. BMC Genom..

[27] Dornelas M.C., Lejeune B., Dron M., Kreis M. (1998). The Arabidopsis SHAGGY-related protein kinase (ASK) gene family: Structure, organization and evolution. Gene.

[28] Wang M., Yue H., Feng K., Deng P., Song W., Nie X. (2016). Genome-wide identification, phylogeny and expressional profiles of mitogen activated protein kinase kinase kinase (MAPKKK) gene family in bread wheat (*Triticum aestivum* L.). BMC Genom..

[29] Youn J.-H., Kim T.-W. (2015). Functional insights of plant GSK3-like kinases: Multi-taskers in diverse cellular signal transduction pathways. Mol. Plant.

[30] Hur E.-M., Zhou F.-Q. (2010). GSK3 signalling in neural development. Nat. Rev. Neurosci..

[31] Doble B.W., Woodgett J.R. (2003). GSK-3: Tricks of the trade for a multi-tasking kinase. J. Cell Sci..

[32] Hu Z., Lu S.-J., Wang M.-J., He H., Sun L., Wang H., Liu X.-H., Jiang L., Sun J.-L., Xin X. (2018). A novel QTL qTGW3 encodes the GSK3/SHAGGY-like kinase OsGSK5/OsSK41 that interacts with OsARF4 to negatively regulate grain size and weight in rice. Mol. Plant.

[33] Hu S. (2016). Brassinosteroid and Gibberellin Control of Plant Height Inmaize (*Zea mays* L). Ph.D. Thesis.

[34] Siddiqui H., Hayat S., Bajguz A. (2018). Regulation of photosynthesis by brassinosteroids in plants. Acta Physiol. Plant..

[35] Wang Z.-Y., Bai M.-Y., Oh E., Zhu J.-Y. (2012). Brassinosteroid signaling network and regulation of photomorphogenesis. Ann. Rev. Genet..

[36] Hu W.-H., Yan X.-H., Xiao Y.-A., Zeng J.-J., Qi H.-J., Ogweno J.O. (2013). 24-Epibrassinosteroid alleviate drought-induced inhibition of photosynthesis in Capsicum annuum. Sci. Hortic..

[37] Li X.-J., Guo X., Zhou Y.-H., Shi K., Zhou J., Yu J.-Q., Xia X.J. (2016). Overexpression of a brassinosteroid biosynthetic gene Dwarf enhances photosynthetic capacity through activation of Calvin cycle enzymes in tomato. BMC Plant Biol..

[38] Xia X.-J., Huang L.-F., Zhou Y.-H., Mao W.-H., Shi K., Wu J.-X., Asami T., Chen Z., Yu J.-Q. (2009). Brassinosteroids promote photosynthesis and growth by enhancing activation of Rubisco and expression of photosynthetic genes in Cucumis sativus. Planta.

[39] He J.-X., Gendron J.M., Yang Y., Li J., Wang Z.-Y. (2002). The GSK3-like kinase BIN2 phosphorylates and destabilizes BZR1, a positive regulator of the brassinosteroid signaling pathway in Arabidopsis. Proc. Natl. Acad. Sci. USA.

[40] Dordas C.A., Sioulas C. (2008). Safflower yield, chlorophyll content, photosynthesis, and water use efficiency response to nitrogen fertilization under rainfed conditions. Ind. Crops Prod..

[41] Rahbarian R., Khavari-Nejad R., Ganjeali A., Bagheri A., Najafi F. (2011). Drought stress effects on photosynthesis, chlorophyll fluorescence and water relations in tolerant and susceptible chickpea (*Cicer arietinum* L.) genotypes. Acta Biol. Crac. Ser. Bot..

[42] Bota J., Medrano H., Flexas J. (2004). Is photosynthesis limited by decreased Rubisco activity and RuBP content under progressive water stress?. New Phytol..

[43] Lin M.T., Occhialini A., Andralojc P.J., Parry M.A., Hanson M.R. (2014). A faster Rubisco with potential to increase photosynthesis in crops. Nature.

[44] Peng P., Yan Z., Zhu Y., Li J. (2008). Regulation of the Arabidopsis GSK3-like kinase BRASSINOSTEROID-INSENSITIVE 2 through proteasome-mediated protein degradation. Mol. Plant.

[45] De Rybel B., Audenaert D., Vert G., Rozhon W., Mayerhofer J., Peelman F., Coutuer S., Denayer T., Jansen L., Nguyen L. (2009). Chemical inhibition of a subset of Arabidopsis thaliana GSK3-like kinases activates brassinosteroid signaling. Chem. Biol..

[46] Clough S.J., Bent A.F. (1998). Floral dip: A simplified method for Agrobacterium-mediated transformation of Arabidopsis thaliana. Plant J..

[47] Fu D.Q., Zhu B.Z., Zhu H.L., Jiang W.B., Luo Y.B. (2005). Virus-induced gene silencing in tomato fruit. Plant J..

[48] Arnon D.I. (1949). Copper enzymes in isolated chloroplasts: Polyphenoloxidase in Beta vulgaris. Plant Physiol..

[49] Song J., Zhou Y., Zhang J., Zhang K. (2017). Structural, expression and evolutionary analysis of the non-specific phospholipase C gene family in Gossypium hirsutum. BMC Genom..

[50] Hu B., Jin J., Guo A.-Y., Zhang H., Luo J., Gao G. (2015). GSDS 2.0: An upgraded gene feature visualization server. Bioinformatics.

[51] Wang Y., Tang H., DeBarry J.D., Tan X., Li J., Wang X., Lee T.-H., Jin H., Marler B., Guo H. (2012). MCScanX: A toolkit for detection and evolutionary analysis of gene synteny and collinearity. Nucleic Acids Res..

[52] Krzywinski M., Schein J., Birol I., Connors J., Gascoyne R., Horsman D., Jones S.J., Marra M.A. (2009). Circos: An information aesthetic for comparative genomics. Genome Res..

[53] Lescot M., Déhais P., Thijs G., Marchal K., Moreau Y., Van de Peer Y., Rouzé P., Rombauts S. (2002). PlantCARE, a database of plant cis-acting regulatory elements and a portal to tools for in silico analysis of promoter sequences. Nucleic Acids Res..

[54] Zhang K., Zheng T., Zhu X., Jiu S., Liu Z., Guan L., Jia H., Fang J. (2018). Genome-wide identification of PIFs in grapes (*Vitis vinifera* L.) and their transcriptional analysis under lighting/shading conditions. Genes.

