# Supplementary material for: Functional Characterization of VvSK Gene Family in Grapevine Revealing Their Role in Berry Ripening
Source: Int J Mol Sci. 2020 Jun 18;21(12):4336. doi: 10.3390/ijms21124336 (PMC7352762; doi:10.3390/ijms21124336)
Supplement: Supplementary file 1 [file ijms-21-04336-s001.zip › Supplementary files/Supplementary Figure1.doc]

**Supplemental Figure1.** Amino acid sequence of motifs in MEME analysis. The number of each sequence corresponds to the motifs number.
